# Evaluation of Cardiac Autonomic Modulation Using Symbolic Dynamics After Cardiac Transplantation

**DOI:** 10.21470/1678-9741-2019-0236

**Published:** 2019

**Authors:** Sílvia Cristina Garcia de Moura-Tonello, Vitor Oliveira Carvalho, Moacir Fernandes de Godoy, Alberto Porta, Ângela Merice de Oliveira Leal, Edimar Alcides Bocchi, Aparecida Maria Catai

**Affiliations:** 1 Physiotherapy Department, Cardiovascular Physiotherapy Laboratory (LFCV), Federal University of São Carlos (UFSCar), São Carlos, SP, Brazil.; 2 Physiotherapy Department, Federal University of Sergipe (UFS), São Cristóvão, SE, Brazil.; 3 Cardiology and Cardiovascular Surgery Department, Medical School of São José do Rio Preto (FAMERP), São José do Rio Preto, SP, Brazil.; 4 Transdisciplinary Nucleus for the Study of Chaos and Complexity (NUTEC), Medical School of São José do Rio Preto (FAMERP), São José do Rio Preto, SP, Brazil.; 5 Department of Biomedical Sciences for Health, University of Milan, Milan, Italy.; 6 Department of Cardiothoracic, Vascular Anesthesia and Intensive Care, IRCCS Policlinico San Donato, Milan, Italy.; 7 Department of Medicine, Federal University of São Carlos (UFSCar), São Paulo, Brazil.; 8 Instituto do Coração do Hospital das Clínicas da Faculdade de Medicina da Universidade de São Paulo (InCor HC-FMUSP), São Paulo, SP, Brazil.

**Keywords:** Heart Transplantation, Autonomic Nervous System, Heart Rate Variability, Nonlinear Dynamics

## Abstract

**Objective:**

To characterize the behavior of cardiac autonomic modulation in individuals with different times after orthotopic heart transplantation (HTx) using symbolic dynamics analysis.

**Methods:**

Sixty patients were evaluated after HTx. We recorded their instantaneous R-R intervals (RRi) by cardiac monitor Polar® RS800CX™ (Polar Electro Oy, Kempele, Finland) for 10 minutes. The same sequence of RRi with 256 consecutive beats was used to perform spectral analysis and symbolic dynamics analysis. We used hierarchical clustering to form groups. One-way analysis of variance (ANOVA) (with Holm-Sidak method) or one-way Kruskal-Wallis test (with Dunn´s post-hoc test) was used to analyze the difference between groups. Linear correlation analysis between variables was performed using Pearson’s or Spearman’s tests. *P*-value < 0.05 was considered statistically significant.

**Results:**

The 0V% index increased, the 2UV% index and the normalized complexity index decreased with an increase of HTx postoperative time. There were a negative correlation between complexity indexes and 0V% and a positive correlation between complexity indexes and 2UV%.

**Conclusion:**

Symbolic dynamics indexes were able to show a specific cardiac autonomic modulation pattern for HTx recipients with different postoperative times.

**Table:** 

Abbreviations, acronyms & symbols				
AH	= Arterial hypertension		HTx	= Orthotopic heart transplantation
ANOVA	= Analysis of variance		InCor HC-FMUSP	= Instituto do Coração do Hospital das Clínicas da Faculdade de Medicina da Universidade de São Paulo
ANS	= Autonomic nervous system		LF	= Low frequency
BMI	= Body mass index		LFabs	= Low frequency in absolute value
CAD	= Coronary artery disease		LFnu	= Low frequency in normalized units
FAMERP	= Medical School of São José do Rio Preto		NCI	= Normalized complexity index
HF	= High frequency		RRi	= R-R intervals
HFabs	= High frequency in absolute value		SE	= Shannon entropy
HFnu	= High frequency in normalized units			
HRV	= Heart rate variability			

## INTRODUCTION

Complete cardiac denervation occurs after orthotopic heart transplantation (HTx) and a recent review showed several studies that investigated the cardiac reinnervation of the autonomic nervous system (ANS)^[1]^. The reinnervation of donor sinus node after heart transplantation can occur and it improves over time^[2]^. It is important because it results in clinical improvements in heart transplant recipients, such as exercise tolerance, peak oxygen uptake, heart rate response, ventricular contractile function, and quality of life^[3,4]^.

Heart rate variability (HRV) is a low-cost and noninvasive method to assess cardiac reinnervation in clinical practice^[5]^. Most of the previous studies used linear methods in the time and frequency domain to analyze HRV in heart transplant recipients^[2,6-8]^. Although spectral analysis is widely used, there are methodological limitations, since the interactions of the human organism on the cardiac autonomic control are complex and do not show linearity^[5]^. This is conceptually limited by frequency bands which were defined by convention and practice^[5]^. In addition, when there is an increase of the low frequency (LF), there must be a reduction of the high frequency (HF), and vice versa, and the sum of LF and HF in normalized units is equal to 100%, characterizing reciprocal changes ^[5,9]^. Moreover, the LF in absolute value (LFabs) index shows information on both sympathetic and parasympathetic modulations^[7,10]^. The HF index reflects generally vagal modulation and respiratory influence^[5,9]^.

Nonlinear methodologies as symbolic analysis, normalized complexity index (NCI), and Shannon entropy (SE) of symbolic dynamics have been used to evaluate HRV in several populations^[11-14]^. The symbolic analysis presents four indexes (0V%, 1V%, 2LV%, and 2UV%) and the sum of those results in 100%^[9]^. A previous study using pharmacological administration observed that 0V% reflects exclusively sympathetic modulation; 1V% reflects modulation of both pathways; 2LV% also reflects sympathetic and parasympathetic modulations, but this index presents vagal predominance; and the 2UV% index reflects exclusively parasympathetic modulation^[14]^. Therefore, the symbolic analysis shows the sympathetic and parasympathetic modulations on the heart exclusively by the indexes 0V% and 2UV%, respectively^[14]^. Furthermore, this analysis can provide nonreciprocal information of sympathetic and parasympathetic modulations differently from indexes of spectral analysis^[14]^.

NCI and SE of symbolic dynamics reflect the complexity of HRV^[15]^. SE evaluates the complexity of the distribution of symbolic dynamics patterns. When the patterns are homogeneously distributed, SE is large, and when some patterns are more frequent and others are less frequent or absent, the entropy decrease. NCI, that also is a complexity index, evaluates the regularity of patterns showing that a more regular signal is more predictable and less complex^[15]^. To the best of our knowledge, there are no studies evaluating HRV by symbolic analysis and SE of symbolic dynamics in heart transplant recipients. In this way, the hypothesis of the present study is that the indexes of the symbolic dynamics can be used to characterize the sympathetic and parasympathetic modulations after HTx. Thus, the aim of this study is to characterize the profile of cardiac autonomic modulation in individuals with different times after HTx using symbolic dynamics analysis.

## METHODS

### Study Population

This is a cross-sectional retrospective study that evaluated 60 orthotopic heart transplant recipients. They were recruited at hospitals after they were examined by the medical staff at different HTx postoperative times. The inclusion criteria were stable condition and no tissue rejection detected by endomyocardial biopsy. We excluded heart transplant recipients with pacemaker, heart failure, Chagas disease reactivation, and chronic obstructive pulmonary disease. The present study was approved by the Research Ethics Committee of the Instituto do Coração do Hospital das Clínicas da Faculdade de Medicina da Universidade de São Paulo (InCor HC-FMUSP), under number 0151/10, and of the Medical School of São José do Rio Preto (FAMERP), under number 251021. All the subjects who participated in the study were informed about the experimental procedures and signed a free and informed consent.

### Experimental Procedure

The experimental procedure was performed at InCor HC-FMUSP and at the Hospital de Base of FAMERP. Firstly, patients were placed supine and remained for 20 minutes to stabilize the cardiovascular variables. After that, we started the recording by cardiac monitor Polar® RS800CX™ (Polar Electro Oy, Kempele, Finland) that registered instantaneous R-R intervals (RRi) for HRV analysis during 10 minutes. The evaluators who performed the HRV analysis were blind regarding the patients’ characteristics and postoperative time.

### Heart Rate Variability

The sequence of RRi with 256 consecutive beats and with the greatest stability in the central region of the tachogram was selected for each volunteer. The same sequence selected was used to perform all the analyses.

### Spectral Analysis

Spectral analysis was performed by the autoregressive model^[5]^. The spectral analysis’ indexes evaluated in this study were: HF in absolute value (HFabs) (0.15 to 0.4 Hz) and in normalized units (HFnu), which reflects vagal modulation and respiratory influence, and LFabs (0.04 to 0.15 Hz), which reflects both sympathetic and parasympathetic modulations^[5,10]^, and LF in normalized units (LFnu), which reflects sympathetic modulation^[5]^.

### Symbolic Dynamics

#### Shannon Entropy

The RRi series were quantized into six levels (from 0 to 5). Each RRi was represented by a symbol depending on the level it was. Thereafter, patterns are formed with three symbols and the distribution of these patterns is calculated by SE. When the patterns are uniformly distributed (flat distribution) and the series carries the maximum information, SE is large. However, when the distribution presents very frequent patterns with infrequent or absent patterns, SE is small^[15]^.

#### Symbolic Analysis

The patterns with three symbols are grouped into four families: 0V, 1V, 2LV, and 2UV^[9]^. 0V consists of patterns that have no variation between their symbols, *e.g*., 1,1,1 or 2,2,2; 1V consists of patterns that have one variation, with two equal symbols and one different, *e.g*., 1,2,2 or 3,3,2; 2LV consists of patterns that form an ascending or descending ramp, *e.g*., 1,2,3 ou 3,2,1; and finally, 2UV consists of patterns in which the three symbols form a peak and or a valley, *e.g*., 3,1,2 or 1,3,2. The symbolic analysis is the percentage of patterns in each family. The index 0V% reflects sympathetic modulation, 1V% reflects modulation of both branches of the ANS, 2LV% has vagal predominance, and 2UV% reflects only vagal modulation^[9,16]^.

#### Conditional Entropy

Conditional entropy was described by Porta et al.^[17]^. We used conditional entropy as a complexity index. This index was normalized by SE of the RRi to obtain NCI. NCI ranges from 0 to 1 (null and maximum information, respectively). When NCI is closer or equal to 1, the complexity is higher and the regularity and predictability are lower^[17]^.

### Statistical Analysis

We divided the groups using hierarchical clustering based on the following variables: variance, 0V%, and 2UV%, by Ward’s method with Euclidean distance squared. We observed that most of the heart transplant recipients were grouped according to the postoperative time: Group 1 was constituted of individuals who were assessed between 53 days to 21 months after HTx; Group 2, of those between 28 and 86 months after HTx; and Group 3, of those between 91 to 145 months after HTx. The distribution of data was verified by the Shapiro-Wilk normality test.

To evaluate if individuals with heart failure due to Chagas disease responded differently than others, we divided each group of HTx (Group 1, Group 2, and Group 3) into two groups: with and without Chagas disease. We performed *t*-test or Mann-Whitney U test, depending on the distribution of data, and we did not observe statistically significant differences in all HRV indices. Therefore, we put the Chagas disease patients with others and did the analysis that is described below.

One-way analysis of variance (ANOVA) was used to analyze the difference between groups, and when there were differences, Holm-Sidak method was used. For nonparametric data it was used one-way Kruskal-Wallis test, and Dunn´s post-hoc test was used when there were differences between groups. The linear correlation analysis between variables was performed using Pearson’s test (for parametric variables) and Spearman’s test (for nonparametric variables). *P*-value < 0.05 was considered statistically significant. The data were presented as mean ± standard deviation and median (first and third quartiles). The Sigma Plot software for Windows version 11.00 was used for data analysis.

## RESULTS

We assessed 60 heart transplant recipients (39 men and 21 women). Age, anthropometric characteristics, and previous transplantation morbidity are shown in Table 1. There were significant differences for age between groups, whereas Group1 was younger than other groups. Considering the etiology of heart failure, we divided each group of HTx (Group 1, Group 2, and Group 3) into two groups: with (43.33%) and without (56.67%) Chagas disease before HTx. However, there were no significant differences for the HRV indices studied. Then, we analyzed the pool of data by HTx time.

**Table 1:** Patients' age, gender, anthropometric characteristics, etiology of heart failure prior to cardiac transplant and time of HTx.

Characteristics	Group 1 (n=22)	Group 2 (n=28)	Group 3 (n=10)	*P*-value
Age (years)	45.33 (37.00-54.13)	54.00 (44.17-57.96)*	51.24 (49.48-64.00)*	0.047
Gender	13 men; 9 women	18 men; 10 women	8 men; 2 women	
Weight (kg)	67.18±15.19	69.48±14.77	69.92±13.09	0.865
Height (m)	1.65±0.08	1.62±0.10	1.69±0.11	0.159
BMI (kg/m2)	24.43±5.05	26.64±5.77	24.48±4.98	0.401
**Etiology**				
Chagas disease	27.27% (n=6)	42.86% (n=12)	60% (n=6)	
Chagas + CAD	4.55% (n=1)	-	-	
Chagas + AH	4.55% (n=1)	-	-	
Idiopathic	54.55% (n=12)	17.86% (n=5)	10% (n=1)	
CAD	4.55% (n=1)	14.29% (n=4)	20% (n=2)	
Re-HTx	4.55% (n=1)	-	-	
Valve insufficiency	-	3.57% (n=1)	10% (n=1)	
Hypertension	-	10.71% (n=3)	-	
Hypertrophic cardiomyopathy	-	3.57% (n=1)	-	
Peripartum cardiomyopathy	-	3.57% (n=1)	-	
Pharmacological treatment for cancer	-	3.57% (n=1)	-	
**Time of HTx**	53 days to 21 months	28 months to 86 months	91 months to 141 months	

AH=arterial hypertension; BMI=body mass index; CAD=coronary artery disease; HTx = orthotopic heart transplantation; Re-HTx = heart re-transplantation Data are presented as mean ± standard deviation and median (first quartile-third quartile). The symbol * indicates difference from Group 1.

Patients used the following medications at the moment of the data recording: cyclosporine (83.33%), tacrolimus (31.67%), azathioprine (31.67%), prednisone (55%), diltiazem (56.67%), simvastatin (56.67%), metformin (11.67%), glyciphage (3.33%), insulin (5%), losartan (16.67%), hydralazine (11.67%), mycophenolate (40%), codeine (3.33%), rivotril (3.33%), fluoxetine (3.33%), gabapentin (3.33%), bromazepam (5%), topiramate (3.33%), enalapril (8.33%), imipramine (3.33%), zolpidem (3.33%), sertraline (3.33%), tramadol (6.66%), rapamune (3.33%), acetylcysteine (3.33%), warfarin (3.33%), carbamazepine (3.33%), allopurinol (3.33%), puran T4 (3.33%), hydrochlorothiazide (3.33%), dormec (3.33%), and propranolol (3.33%).

**Fig. 1:**
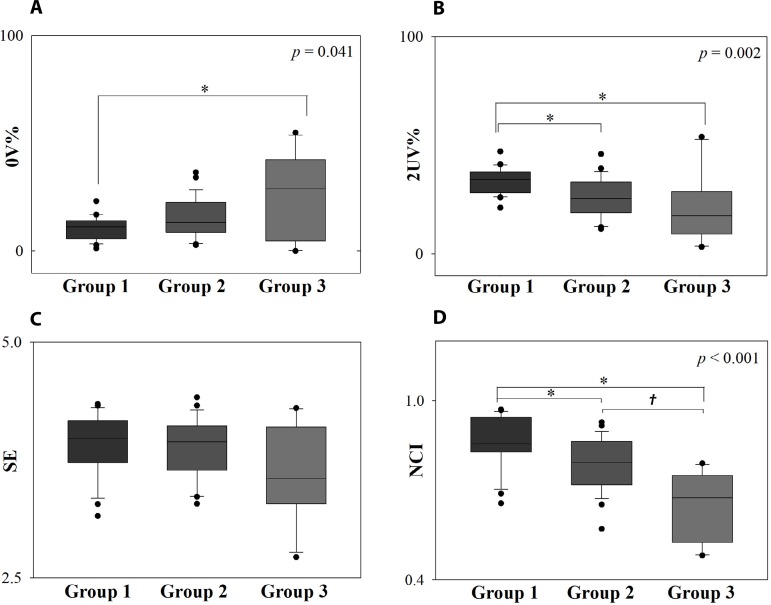
Symbolic analysis and complexity indexes. Box-and-whiskers plots of 0V% (A), 2UV% (B), Shannon entropy (SE) (C), and normalized complexity index (NCI) (D). The symbol * indicates the difference from Group 1 and the symbol † indicates the difference from Group 2. P-value is indicated in each graph when there are differences.

The time and spectral domain indexes of HRV are showed in Table 2. Individuals with a long post-transplant period (Group 3) had higher variance (total variability), LFabs, and HFabs than subjects with a recent HTx postoperative time (Group 1). The difference between groups for symbolic and complexity indexes (SE and NCI) are shown in Figure 1. Group 3 presented higher 0V% value than Group 1. Regarding 2UV%, Groups 2 and 3 presented lower values than Group 1. There was no difference between groups for 1V% (Group 1: 41.30 ± 7.68; Group 2: 45.06 ± 7.26; Group 3: 41.61 ± 10.65; *P*=0.222) and 2LV% indices (Group 1: 11.96 [9.41-17.65]; Group 2: 13.33 [6.08-20.78]; Group 3: 7.06 [3.14-14.12]; *P*=0.201). There was no difference between groups for SE. The groups were different for NCI; Group 1 had higher value than Groups 2 and 3, and there was a difference between Groups 2 and 3.

**Table 2:** Linear analysis of heart rate variability.

	Group 1 (n=22)	Group 2 (n=28)	Group 3 (n=10)	*P*-value
Mean (ms)	695.59±83.04	714.59±86.17	759.44±117.21	*P*=0.201
Variance (ms^2^)	3.69 (2.30-9.21)	15.89 (8.63-25.00)*	85.50 (67.98-101.19)*†	*P*<0.001
LFabs (ms^2^)	0.12 (0.00-0.41)	0.63 (0.14-1.73)	11.76 (1.08-27.96)*	*P*<0.001
HFabs (ms^2^)	1.97 (0.93-4.93)	4.05 (2.30-9.91)	8.81 (5.53-38.04)*	*P*=0.004

HFabs=high frequency in absolute value; LFabs=low frequency in absolute value Values are presented as mean ± standard deviation and median (first quartile-third quartile). The symbol * indicates the difference from Group 1 and the symbol † indicates the difference from Group 2.

**Fig. 2:**
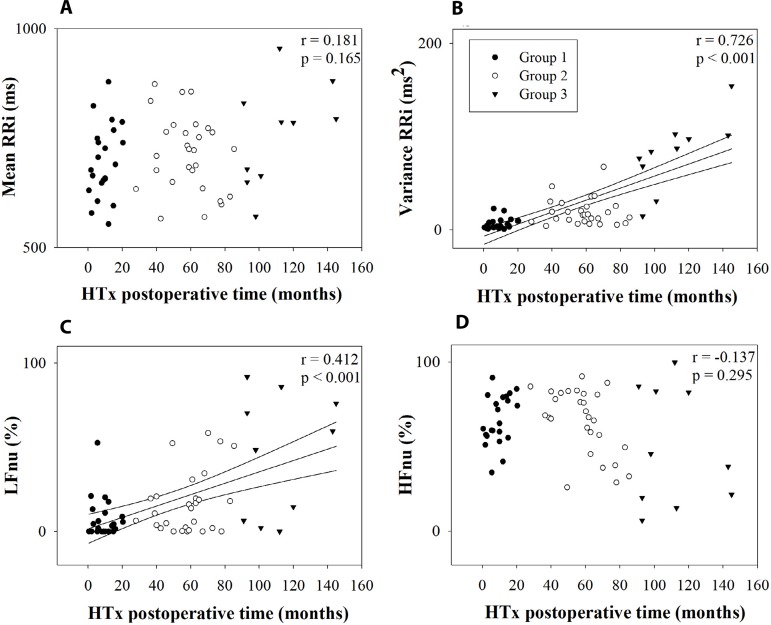
Relation between linear analysis and heart transplantation (HTx) postoperative time. Linear regression and its 95% confidence interval of variance (B) and low frequency in normalized unit (LFnu) (C). Group 1 is represented by ●, Group 2 is represented by ○, and Group 3 is represented by ▾. HFnu=high frequency in normalized units; LFnu=low frequency in normalized units; RR=R-R intervals.

**Fig. 3:**
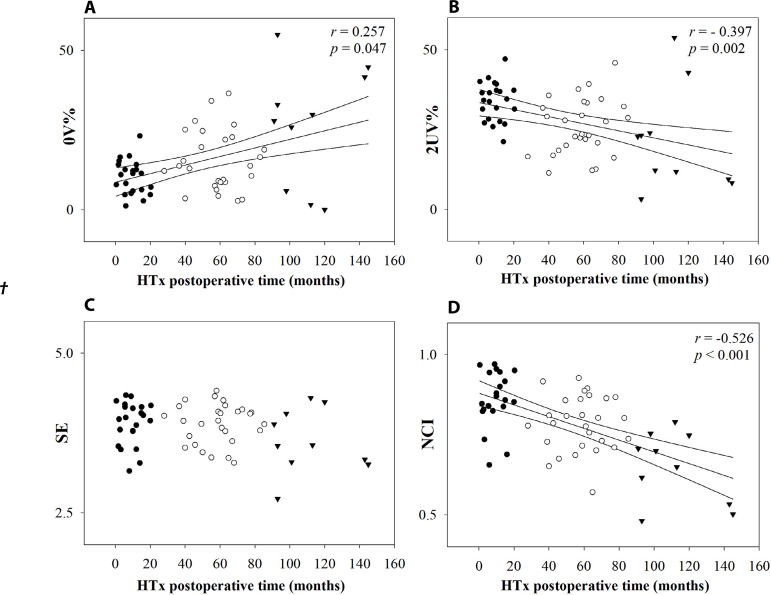
Relation between symbolic dynamics indexes and heart transplantation (HTx) postoperative time. Linear regression and its 95% confidence interval of 0V% (A), 2UV% (B), Shannon entropy (SE) (C), and normalized complexity index (NCI) (D). Group 1 is represented by ●, Group 2 is represented by ○, and Group 3 is represented by ▾ in graphs a, b, c and d. P-value is indicated in each graph when there are differences.

Figures 2 and 3 illustrate the relation between HTx postoperative time with time and spectral domain indexes and symbolic dynamics indexes, respectively. There was an increase in variance and LFnu with the HTx postoperative time (strong and weak positive correlation, respectively). The symbolic analysis indexes also had correlation with HTx postoperative time. 0V% increased (weak positive correlation [Figure 3A]) while 2UV% decreased with the increase of post-transplant time (weak negative correlation [Figure 3B]). Relative to the complexity indexes, only NCI presented decrease with HTx postoperative time (moderate negative correlation [Figure 3D]).

## DISCUSSION

The main finding of the present study is that by symbolic and complexity analyses it is possible to observe an autonomic modulation profile for subjects with different HTx postoperative times. This profile was characterized by increased 0V% and decreased 2UV% and decreased complexity (NCI), with an increase of time after HTx. In addition, symbolic dynamics complemented the frequency domain analysis showing the reduction of 2UV% and NCI after HTx.

Using the results of the symbolic analysis (0V% and 2UV%), we performed hierarchical clustering to try to form groups, and we observed the presence of three patterns according to the HTx postoperative time. Similarly, a previous study assessing heart transplant recipients (1 to 180 months of postoperative time) using spectral analysis indexes performed hierarchical clustering and founded three groups, concluding that clustering is a useful tool to evaluate the behavior of cardiac reinnervation by HRV[18]. In the present study, we observed a cardiac autonomic modulation profile due the similarity of HRV results (0V% and 2UV%) in individuals with 53 days to 21 months (Group 1: 9.28 ± 5.79 months), 28 to 86 months (Group 2: 58.39 ± 14.56 months), and 91 to 145 months (Group 3: 110.90 ± 19.95 months) of transplantation postoperative time.

Despite the etiology of HTx was not considered by hierarchical clustering analysis, in the present study it was observed that 43.33% of individuals had Chagas cardiomyopathy. The literature shows that individuals with Chagas disease may have autonomic dysfunction with neuronal lesions of ANS fibers^[19]^. Therefore, we divided each group of HTx (Group 1, Group 2, and Group 3) into two groups, with and without Chagas disease, to evaluate if there was any influence of this disease after transplantation. However, we did not observe significant differences in all HRV indices studied. So, we jointly analyzed the data by group with and without Chagas disease.

In relation to the cardiac autonomic modulation profile in individuals with different times after HTx, by symbolic dynamics analysis, it was characterized by increased cardiac sympathetic modulation observed by difference in 0V% between Groups 1 and 3 (Figure 1A) and by relation between 0V% and HTx postoperative time (Figure 3A). This result was confirmed when we observed LFnu of the present study. The majority of the recipients of Groups 1 and 2 presented low values of LFnu, while 60% of Group 3 presented similar values to that found in healthy subjects in a previous study (Figure 2C)^[13]^. We emphasize that normalized units are expressed as a percentage, but when we observed LFabs we noticed that the recipients presented very reduced values compared to healthy individuals reported in literature (in the present study, the mean of all groups was 4.55 ± 11.56 ms^2^ *vs*. 545 ± 495 ms^2^ for the healthy subjects^[13]^). To the best of our knowledge, there are no studies using symbolic analysis to evaluate cardiac autonomic modulation in heart transplant recipients, but considering the spectral analysis, our results corroborate with a previous study^[20]^.

In addition, we observed the possible sympathetic reinnervation and its improvement by positive and weak correlation between 0V% and HTx postoperative time, *i.e*., possibly the sympathetic modulation increases over time. This result corroborated with a previous study using frequency domain analysis that reported that the sympathetic reinnervation of the donor heart sinus node occurs about one year after transplantation^[21]^.

The profile was characterized also by decreased 2UV% with increase of HTx postoperative time (negative and weak correlation [Figure 3B]). In addition, there were differences between Group 1 *vs*. 2, and Group 1 *vs*. 3 (Figure 1B). Despite this index indicates parasympathetic autonomic modulation^[14]^, we cannot infer that there is a reduction of the cardiac parasympathetic modulation in this population, because Group 1 was constituted by individuals with recent HTx postoperative time (*i.e.*, recently denervated) and presented higher values than other groups.

Considering the findings of the spectral analysis of this study, which was carried out in the same stationary section that the symbolic analysis, we observed that there was an increase in HFabs with significant difference between Groups 1 and 3, whereas for HFnu there was no difference. This result corroborates with a previous study that observed an increase in HFabs when it compared recent postoperative period and follow-up after 10 years, but HFnu did not change^[6]^. However, van De Borne et al.^[20]^ (2001) observed a reduction of HFnu in subjects with late HTx postoperative time (103 to 163 months) compared to those with recent postoperative time (1 to 14 months), showing similar behavior to variable 2UV% in the present study. It is noteworthy that the values of HF band are similar to those of previous studies^[6,20]^. The HTx recipients, regardless of postoperative time, presented lower values of HF in absolute values (ms^2^) than, for example, individuals with coronary artery disease with and without acute myocardial infarction and healthy subjects evaluated in a previous study^[13]^.

Some authors have reported that the HF band may not be such a reliable marker of vagal reinnervation and modulation in patients submitted to HTx because it reflects nonautonomic mechanisms, such as respiration or atrial stretch due alterations in venous return^[20,22]^. Possibly the 2UV% index in Group 1 is also reflecting nonautonomic mechanisms such as those described for HF band. Compared to Group 2 (4.87 ± 3.88 years of HTx postoperative time) and Group 3 (9.24 ± 1.58 years of HTx postoperative time), the results probably reflect a mix of nonautonomic mechanisms and parasympathetic modulation, since previous studies using HRV analysis reported that parasympathetic reinnervation occurs after four years of HTx^[21]^, while Lee et al.^[2]^(2016) concluded that it seems to begin less than one year after bicaval heart transplantation. Imamura et al.[8] (2014) showed that it gradually occurs in less than six months after heart transplantation using modified bicaval technique especially in recipients with lesser time of cardiopulmonary bypass. However, according to Cornelissen et al.^[6]^ (2012), the reinnervation process is still controversial and the presence of frequency components suggests partial functioning of the cardiac autonomic modulation. So, it seems that a process of parasympathetic reinnervation also had occurred in the present study, as detected by symbolic analysis.

In addition, we evaluated the complexity signal by symbolic dynamics through SE and NCI. There was no difference relative to distribution of patterns (SE), but there was a difference relative to the regularity of patterns (NCI). These results suggest that probably NCI may have more sensitivity than SE for this population. A previous study that evaluated a healthy population also observed differences just in NCI, when it compared elderly with young subjects, and SE did not present any difference between groups^[12]^. Thus, the cardiac autonomic modulation profile also was characterized by decrease of complexity (NCI) with increase of time after HTx. Moreover, NCI had a negative and moderate correlation with HTx postoperative time showing an increase of regularity and, consequently, reduction of complexity of signals over time.

The symbolic dynamics analysis showed to be important and complementary to linear analysis to evaluate recipients after HTx. This analysis demonstrated the reduction of NCI and 2UV% with increase of HTx postoperative time, even though the 2UV% for this population probably reflected the mixed action of cardiac vagal modulation and nonautonomic mechanisms. Moreover, the decrease of NCI, usually associated with a sympathetic activation^[15,17]^, could be the consequence of the reactivation of a causal link between arterial pressure and heart period that was demonstrated to become more and more active with HTx postoperative time^[23]^. These results may be due to the probable lifetime of the organ and the patient submitted to transplantation, since the median survival of patients is 11 years after heart transplantation^[24]^. Other factor that may have influenced these results was the patients’ age, since Group 1 was younger than other groups and HRV and NCI decreases with the ageing^[25]^. However, we observed that there was an autonomic modulation profile for subjects with different HTx postoperative times.

The clinical relevance of these findings is the presence of cardiac autonomic profile after HTx, supporting early cardiorespiratory rehabilitation protocols for this population in order to benefit the autonomic modulation and tolerance to physical exercise and, consequently, the quality of life.

Differently from the present study, Takakura et al.^[26]^ (2017) observed using other nonlinear analysis (average diagonal length and sample entropy) higher predictability and lower complexity in a recent transplant group when compared with a group with a longer HTx postoperative time. Makowiec et al.^[27]^ (2013) used the entropy of transition and suggested that there is an increase in entropy with the increase in the postoperative time of cardiac transplantation because this was the behavior presented by the majority. However, the authors showed a figure with some individuals that presented the same behavior of the present study, *i.e*., reduction of complexity observed by entropy^[25]^. This difference can be explained by the reduced sample size (14 patients submitted to cardiac transplantation) and by the smaller postoperative time (maximum 36 months) when compared with the present study. Thus, studies using nonlinear analysis of HRV are necessary and important to evaluate this population.

## CONCLUSION

Symbolic dynamics indexes were able to characterize a cardiac autonomic modulation profile for heart transplant recipients with different postoperative times. This profile was characterized by increased 0V%, decreased 2UV%, and a decrease of complexity (NCI) with an increase of time after HTx, probably due the organ and patient lifetimes after HTx.

**Table:** 

Authors' roles & responsibilities	
SCGMT	Analysis and interpretation of data for the work; drafting the work and revising it critically for important intellectual content; agreement to be accountable for all aspects of the work in ensuring that questions related to the accuracy or integrity of any part of the work are appropriately investigated and resolved; final approval of the version to be published
VOC	Substantial contributions to the conception and design of the work; acquisition of data for the work; revising the manuscript critically for important intellectual content; final approval of the version to be published
MFG	Acquisition of data for the work; revising the manuscript critically for important intellectual content; final approval of the version to be published
AP	Revising the manuscript critically for important intellectual content; final approval of the version to be published
AMOL	Revising the manuscript critically for important intellectual content; final approval of the version to be published
EAB	Substantial contributions to the conception and design of the work; revising the manuscript critically for important intellectual content; final approval of the version to be published
AMC	Substantial contributions to the conception and design of the work; interpretation of data for the work; revising the manuscript critically for important intellectual content; agreement to be accountable for all aspects of the work in ensuring that questions related to the accuracy or integrity of any part of the work are appropriately investigated and resolved; final approval of the version to be published
